# Effect of Dietary Fiber Sources on In-Vitro Fermentation and Microbiota in Monogastrics

**DOI:** 10.3390/ani10040674

**Published:** 2020-04-13

**Authors:** Asavela Ngalavu, Hailong Jiang, Saeed El-Ashram, Guillermo Tellez-Isaias, Mohammed Hamdy Farouk, Pakama Siphelele Nyingwa, Adams Seidu, Thobela Louis Tyasi

**Affiliations:** 1 College of Animal Science and Technology, Jilin Agricultural University, Changchun 130118, China; asavelangalavu@ymail.com (A.N.); pakamanyingwa@gmail.com (P.S.N.); 2 Jilin Provincial Key Lab of Animal Nutrition and Feed Science, Jilin Agricultural University, Changchun 130118, China; 3 College of Life Science and Engineering, Foshan University, 18 Jiangwan street, Foshan 528231, China; saeed_elashram@yahoo.com; 4 Faculty of Science, Kafrelsheikh University, Kafr el-Sheikh 33516, Egypt; 5 Department of Poultry Science, University of Arkansas, Fayetteville, AR 72701, USA; gtellez@uark.edu; 6 Department of Animal Production, Faculty of Agriculture, Al-Azhar University, Nasr City, Cairo 11884, Egypt; 7 Department of Veterinary and Biomedical Sciences, South Dakota State University, Brookings, SD 57007, USA; adamyazori@gmail.com; 8 Department of Agricultural Economics and Animal Production, University of Limpopo, Polokwane 0727, South Africa; louis.tyasi@ul.ac.za

**Keywords:** alfalfa, cornstalk, gut health, rice straw

## Abstract

**Simple Summary:**

The recent findings on the role played by fiber constituents during digestion remain controversial, while the impact of high fiber feeds on digestion depends on the type, form, and the level of inclusion of fiber in the diet. This study aims to determine the effect of different fiber forms in pig diets on intestinal nutrient digestibility, hindgut fermentation, and microbial community composition. We formulated four experimental groups (group A = alfalfa, group B = corn stalk, group C = rice straw, and group D = controls) to evaluate the effect of dietary fiber sources on in-vitro fermentation and microbiota. Cornstalk showed a high digestibility when compared to alfalfa and rice straw. Greater digestibility of corn stalk than rice straw and alfalfa in these results may be due to the lower crude fiber content in corn stalk when compared to alfalfa and rice straw. In conclusion, corn stalk dietary fiber improved dry matter digestibility in simulated in vitro digestion experiments, whereas rice straw fiber improved volatile fatty acid content in *simulated* in vitro digestion and fermentation efficiency. Furthermore, alfalfa fiber improved the thickness of *Firmicutes* and *Lactobacillus* supernatant in simulated in vitro digestion treatments.

**Abstract:**

Feed fiber composition is usually considered as one of the factors that have an impact on digestive tract microbiota composition. The investigations on the level of fermentation and in-vitro digestibility of different fibers are not well understood. The aim of the current study is to determine the effect of different fiber sources on intestinal nutrient digestibility, hindgut fermentation, and microbial community composition under in vitro conditions using pigs’ hindgut as a model. The experimental treatment diets contained alfalfa hay, cornstalk, and rice straw. Cornstalk treatment displayed higher digestibility compared to alfalfa hay and rice straw; similar results were observed with in-vitro digestibility using intestinal digesta. *Firmicutes* were the most abundant phyla (*Firmicutes* = 89.2%), and *Lactobacillus* were the prominent genera (75.2%) in response to alfalfa compared to rice straw and cornstalk treatments. In simulated in-vitro digestion, corn stalk fiber improved dry matter digestibility; rice straw fiber improved volatile fatty acid content and fermentation efficiency. Alfalfa fiber improved the thickness of deposited *Firmicutes* and *Lactobacillus*.

## 1. Introduction

The determination of fiber digestibility by in-vitro fermentation of feedstuff is applied in research and commercially to evaluate the nutritional value of fibrous feeds for livestock [1,2]. Although in-vivo determination fiber digestibility is regarded as standard for digestibility evaluation, it remains time consuming, expensive, and labor intensive [3]. Therefore, in-vitro techniques are highly used and are often correlated with in-vivo estimates [4]. In-vitro digestion models are mostly used to investigate structural changes, digestibility, and liberation of feed components under stimulated gastrointestinal conditions. 

According to Williams et al. [5], dietary fiber contains non-digestible constituents, which physiologically influence digestion by rearranging digesta, modulating digestion processes, and acting as the main substrate for microbial fermentation. Feed fiber composition is usually considered as one of the factors that have an impact on digestive tract microbiota composition and the movement of dietary fiber constituents, leading to changes in the composition of microbiota [6]. However, the current known role of fiber constituents during digestion remains controversial, while the impact of high fiber feeds on digestion depends on the type, form, and the level of inclusion of fiber in diet [7]. Previous research has been performed on the digestibility of dietary fiber using in-vivo and in-vitro techniques. Recently, Zhao et al. [8] have studied the effect of wheat bran, corn bran, sugar beet pulp, oat bran, soybean hulls, or rice bran on ileal digestibility and the levels of volatile fatty acids in growing pigs; however, investigations on the level of fermentation, in-vitro digestibility and the microbiome composition of different fibers such as rice straw, alfalfa hay, corn stalk are not well understood. 

Hence, we hypothesize that different levels and sources of dietary fibers influence changes in short-chain fatty acid levels and optimal digestibility. Furthermore, there is an association between diet and concentrations of different species and genera of bacterial communities in the cecum. The objective of this study is to determine the effect of different fiber forms on intestinal nutrient digestibility, hindgut fermentation, and microbial community composition under in vitro conditions using pigs’ hindgut as a model.

## 2. Materials and Methods 

### 2.1. Study Site

The study was conducted at the Animal Nutrition and Feed Science Department Laboratories, Jilin Agricultural University, Jilin Province, Changchun City, P.R. China. Jilin Agricultural University is situated at the latitude of 43°42′ N and the longitude 126°12′ E, while Changchun City is located at the latitude of 43º 88′ N and the longitude of 125° 35′ E. 

### 2.2. Ethical Considerations

To conform to national and international research standards, this study was conducted according to ethical principles governing the usage of animal samples in research studies of this nature. Permission to use samples was also acquired from the Ethical Clearance Committee of Jilin Agricultural University.

### 2.3. Feed Collection and Preparation

Samples of cornstalk, alfalfa hay, and rice straw were collected from Dao Xi farm in Changchun City. After removing the leaves, feeds were oven-dried at a temperature of 90 °C until a constant weight was attained. After complete drying, corn stalk, alfalfa hay, and rice straw were cut into smaller pieces (5–10 cm) then crushed into a fine powder to permit passing through a ≥3.5-cm mesh sieve.

### 2.4. Biological Sample Collection and Preparation

Hindgut samples (*n* = 4) were collected immediately after slaughter from the commercial abattoir in Changchun City, China. Samples were stored in a cooler box containing ice tubes from the abattoir to the laboratory. Animal management, diet, age, sex, and a breed of pigs slaughtered for sample collection were not considered. Immediately after arrival at the laboratory, samples were cut into two cross-sectional areas in the cecum, and approximately 120 g of digesta was drained off into sterile test tubes and immediately frozen at −80 °C for further analysis.

### 2.5. Experimental Design

The experimental design was a complete randomized design. Four experimental groups were used in this study (group A = alfalfa; group B = cornstalk; group C = rice straw; and group D = had no feed sample control). The experimental treatment diets contained alfalfa hay, cornstalk, and rice straw. The experiments were replicated 3 times.

### 2.6. Chemical Analysis

The chemical analysis was conducted in the laboratory at room temperature and normal room relative humidity. All feed samples were analyzed for crude protein (CP), ether extract (EE), ash content, and percent of moisture according to the AOAC [9]. Crude fiber (CF) was analyzed according to the Van Soest [10] method. Nitrogen free extract (NFE) was calculated according to the AOAC [9] procedure, as depicted in Table 1.

### 2.7. In-Vitro Digestibility of Dry Matter (DM)%

The first step experiment mimicked the gastric stage of digestion. The experiment was conducted in-vitro using 3 steps according to the Boisen and Fernández [11] method, as described by Jaworski [12]. Feed samples were weighed, and 0.5 g of each sample was placed into 125 mL Erlenmeyer flasks, excluding the control group. Twenty-five mL of phosphate buffer (0.1 M), a pH 6.0, and 10 mL (0.2 M) hydrochloric acid were added to each flask, and flasks were mixed continuously using a magnetic stirrer. The pH of the solution was maintained at 2 ± 0.01 by adding 1 M of hydrochloric acid. One milliliter of fresh pepsin solution (25 mg of pepsin/mL; P7000, Sigma Aldrich, St. Louis, MO, USA) was added to each flask. Samples were incubated in a 39 °C shaking water bath for 2 h. 

The second stage of the experiment mimicked digestion in the small intestine. The soluble fiber was not removed through filtration and was maintained in the undigested residue once the small intestine digestion stage was done. 

After 2 h, 10 mL of phosphate buffer (0.2 M), pH 6.8 and 5 mL sodium hydroxide (0.6 M) were added to each flask. The pH was maintained at 6.8 ± 0.01 by adding 1 M of NaOH. One milliliter of freshly prepared pancreatin solution was added to each flask (100 mg of pancreatin/mL; P1750, Sigma Aldrich, St. Louis, MO, USA). Samples were incubated in a shaking water bath at 39 °C for 4 h [13].

The third and last step prompted the fermentation processes in the large intestine. Gooch crucibles were weighed for the determination of the digestibility step.

After 4 h, 10 mL of 0.2 M EDTA solution was added to each flask. About 30% acetic acid was used to adjust the pH to 4.8 ± 0.01, and 0.5 mL of Viscozyme (Viscozyme L V2010, Sigma-Aldrich, St. Louis, MO, USA) was added to each flask. The flasks were incubated in a 39 °C shaking water bath for 18 h. To discover digestibility, residues that were left in the flasks were filtered in Gooch crucibles that contained celite 545 (0.400 g ± 5 mg; Sigma Aldrich, St. Louis, MO, USA). The undigested residues that were left in the crucibles were washed twice with 10 mL of 96% ethanol and 10 mL of 99.5% acetone. The crucibles were oven-dried for 2 h at 130 °C. After cooling in the desiccator, crucibles were weighed to measure total tract dry matter (DM) residues [11]. Two extra flasks that were used as controls were included to correct the final DM weight of the residues. 

The in-vitro digestibility of DM was calculated using the following equation [14]: *In vitro digestibility of DM*% = (*Sample DM* − (*Residue DM* − *Blank DM*))/*Sample DM* × *100*.

### 2.8. In Vitro Digestibility Using Intestinal Digesta

Feed samples were weighed, and 0.5 g of each sample was deposited into 50 mL test tubes, excluding the control group. About 7.5 g of digesta was added to each tube, then tubes were tightly closed with a screw cap lid, and samples were placed and incubated in a shaking water bath that was heated at 39 °C for 18 h. After 18 h, samples were centrifuged at 1200× *g* at 4 °C for 30 min. Supernatants were removed and stored at −80 °C for volatile fatty acid (VFA) and microbial composition analysis, whereas precipitates were dried and analyzed for fiber. 

For the determination of digestibility, CF was analyzed on undigested residues using the Van Soest [10] method. Briefly, residual feed samples were weighed and added into a weighed filter bag. Filter bags were sealed with a sealing machine. Blank filter bags were added as a control. Samples were analyzed using the ANKOM 2000i analyzer. After analysis, samples were washed with acetone and ethanol, then air-dried overnight. CF results were calculated using the ANKOM data spreadsheet.

In vitro digestibility of fiber was calculated using the classical formula described by Perez et al. [15] as the following:

CF digestibility = (Original sample × CF% before digestion) – (Digested sample × CF% after digestion)/(Original sample * CF before digestion).

### 2.9. Volatile Fatty Acid Analysis

Volatile fatty acid standard solution was prepared, which contained 344 µL acetic, 373 µL propionic acid, and 184 µL n-butyric acid in 100 mL volumetric flasks and mixed with ultra-pure water to make 60 mmol/L of actic acid, 50 mmol/L propionic acid and 20 mmol/L mixed standard solutions. Pig digesta fluid was filtered through 4 layers of sterilized gauze, and 1.2 mL of the filtrate was added in centrifuge tubes and centrifuged at 10,000 r/min for 10–15 min. Approximately 1 mL of the supernatant was added to a centrifuge tube, and 0.2 mL of 25% metaphosphoric acid solution was added in a ratio of 5:1 and uniformly mixed with a vortex shaker. Samples were centrifuged at 10,000 rpm/min for 10–15 min and tested. The chromatogram of the VFA samples was obtained, and the samples were calculated by the peak area external standard method to determine the content of volatile fatty acids. To determine the standard curve, the mixed standard solutions were diluted to prepare a series of standard solutions of different concentrations. The mixed standard solution (1 mL) was added into centrifuge tubes with 0.2 mL of 25% metaphosphoric acid solution, mixed evenly with a vortex shaker, and centrifuged at 1000 rpm for 15 min. The supernatant (1 mL) was placed into the gas chromatography with a microsyringe for analysis. The peak area was recorded to determine the standard curve.

### 2.10. Preparation of 16S rRNA Samples

Samples for the 16S rRNA experiment were only obtained from pig’s digesta post in vitro digestion experiment. Samples were prepared as explained in Section 2.8.

### 2.11. Total DNA Extraction, (PCR) Amplification, and High-Throughput Sequencing

As previously described [16,17,18], next-generation sequencing (NGS) and library preparations were carried out at Shanghai Personal Biotechnology Co., Ltd., Shanghai, China, by an Illumina MiSeq (Illumina, San Diego, CA, USA). DNA (30–50 ng) was extracted using TIANGEN DP336 genomic-DNA extraction kits (Biotech Co. Ltd., Beijing, China) and quantified with a Qubit 2.0 Fluorometer (Invitrogen, Carlsbad, California, US) to advance amplicons (400–450 bp) using a MetaVx Library Preparation Kit (Shanghai Personal Biotechnology Co., Ltd., Shanghai, China) [16,17,18]. The quality of DNA extraction was detected by 0.8% agarose gel electrophoresis, and the DNA was quantified using a UV spectrophotometer. The amplification system and procedure of PCR were as follow: 5×reaction buffer 5 μL, 5×GC buffer 5 μL, dNTP (2.5 mM) 2 μL, forward primer (10 uM) 1 μL, reverse primer (10 uM) 1 μL, DNA Template 1 μL, ddH2O 9.75 μL, DNA polymerase 0.25 μL. The following steps were performed in PCR amplification: initial denaturation at 98 °C for 5 min, 25 cycles at 98 °C for 30 s, 52 °C for 30 s and 72 °C for 1 min, and a final extension at 72 °C for 5 min at 12 °C. The PCR products were purified with Agencourt AMPure XP magnetic beads (Beckman Coulter, Brea, CA, USA) on the DynaMag-96 Side Magnet (Thermo Fisher Scientific, Landsmeer, the Netherlands). The PCR products were quality controlled with TapeStation (Agilent Technologies, Santa Clara, CA, USA), and the final DNA concentrations of the purified products were measured with a Qubit 2.0 fluorometer (Thermo Fisher Scientific, Waltham, MA, USA).

The PCR amplification product was detected by 2% agarose gel electrophoresis, and the target fragment was cut and recovered. The recovery was performed using the gel recovery kit from AXYGEN. The V3 and V4 hypervariable regions of prokaryotic 16S ribosomal RNA (rRNA) were selected for inducing amplicons, following taxonomic analysis on each 40 ng DNA sample. Genewiz has formulated a panel of proprietary primers aimed at relatively conserved regions flanking the V3 and V4 hypervariable regions of bacteria and Archaea 16S rDNA. The V3–V4 region of bacteria 16S rRNA gene was amplified by PCR using primers 338F (5′-ACTCCTACGGGAGGCAGCA-3′) and 806R (5′-GGACTACHVGGGTWTCTAAT-3′). The first-round PCR products were used as templates for a second round of amplicon enrichment by PCR. We used MiSeq sequencer for 2 × 300 bp double-end sequencing. The corresponding reagent is the MiSeq Reagent Kit V3 (600 cycles).

Before sequencing on the machine, we performed quality control on the library on an Agilent Bioanalyzer, using the Agilent High Sensitivity DNA Kit. Qualified libraries have only one peak and no adapters. Due to the limitation of MiSeq sequencing read length, and in order to ensure the sequencing quality, the optimal sequencing insert range was 200–450 bp. Indexed adapters were added at the same time to the ends of the 16S rDNA amplicons to produce indexed libraries ready for downstream NGS on the Illumina MiSeq. DNA libraries were validated by an Agilent 2100 Bioanalyzer (Agilent Technologies), quantified by Qubit 2.0 Fluorometer (Invitrogen), multiplexed, and loaded onto the Illumina MiSeq as per manufacturer’s instructions. NGS was performed by a 2 × 300 paired-end (PE) configuration [19], and image analysis was conducted and base calling with the MiSeq Control Software (MCS) embedded in the MiSeq instrument [20]. The amplicon sequence data were deposited in the National Center for Biotechnology Information (NCBI) with accession number SRR2579284 and ERS2011824.

### 2.12. Sequence Analysis

The QIIME data analysis package was used for 16S rRNA data analysis [21]. The forward and reverse reads were joined and assigned to samples based on barcode and truncated by cutting off the barcode and primer sequences. Quality filtering on joined sequences was performed, and any sequence that did not fulfill the following criteria was removed: sequence length < 200 bp, no ambiguous bases, and mean quality score ≥ 20. Sequences were compared with the reference database (RDP Gold database) using the UCHIME algorithm to detect chimeric sequences, which were then removed. The effective sequences were used in the final analysis. The UCLUST sequence alignment tool was used to merge and divide operational taxonomic units (OTUs) according to 97% sequence similarity [22].

The Ribosomal Database Program (RDP) classifier was used to assign a taxonomic category to all OTUs at a confidence threshold of 0.8. The RDP classifier uses the Silva 119 database, which has taxonomic categories predicted to the species level. Sequences were rarefied prior to the calculation of alpha and beta diversity statistics. Alpha diversity indexes were calculated in QIIME from rarefied samples using the Shannon index for diversity, and the Chao1 index for richness. Beta diversity was calculated using weighted and unweighted UniFrac and principal coordinate analysis (PCoA). The unweighted pair group method with arithmetic mean (UPGMA) tree from the beta diversity distance matrix was built. The species accumulation curves are similar to the sparse curves, which are used to measure and predict the increase of species richness with the increase of sample size. They are widely used to judge whether the sample size is sufficient and estimate community richness [23]. Through the species accumulation curve, we can not only estimate whether the sample size is enough to reflect the diversity differences between different communities but also roughly estimate the upper limit of community diversity (generally analyzed when the sample size is more than 10).

### 2.13. Statistical Analysis

Data were analyzed using one-way analysis of variance (ANOVA) with Duncan multiply range test for comparing mean differences. Statistical package for social sciences (version 16.0 for Windows, SPSS Inc., Chicago, IL, USA) was used. Results were expressed as mean ± standard error of the mean (SEM). The significance level of the study was set at α (alpha) = 0.05. 

OTU classification results, principal component analysis (PCA), heat mapping, and the partial least squares discriminant analysis (PLS–DA) discriminant model were analyzed and constructed by R software (version 3.1–137) [24]. Alpha and beta diversities were uniformly calculated, statistically analyzed, and randomized at 90% using QIIME software version 1.8.0 [21]. The statistical algorithm of Metastats was analyzed using Mothur software (Version 1.35.1) [25]. The difference in quantity (absolute abundance) was analyzed using pairwise comparison. The LEfSe was analyzed and visualized by the Galaxy online analysis platform (http://huttenhower.sph.harvard.edu/galaxy/).

## 3. Results

### 3.1. In-Vitro Digestibility of DM%

The results of the in-vitro digestibility of DM are shown in Table 2. Cornstalk treatment had shown higher digestibility (*p* < 0.05) of DM% (22.61) when compared to rice straw (18.36) and alfalfa (19.72) treatments. DM% from alfalfa treatment was not significantly different from that of rice straw and corn stalk (*p* < 0.05) but highly significant from the control group (*p* > 0.05). However, controls were significantly different from all treatment groups (*p* < 0.05).

Table 3 compares the Pearson correlation between treatments at *p* < 0.01. There is a positive correlation between alfalfa and cornstalk and a negative correlation between alfalfa and rice straw, while rice straw correlates positively with cornstalk.

### 3.2. In-Vitro Digestibility Using Intestinal Digesta

Digestibility response in Table 2 demonstrated that alfalfa treatment was not significantly different from rice straw and cornstalk treatments, whereas the significant difference was observed between the three treatments and the control group. 

Rice straw treatment was significantly different from cornstalk and untreated control groups, and cornstalk treatments differed significantly from the controls. However, these results showed no effect on the controls. 

Table 4 shows the Pearson correlation between treatments at *p* < 0.01. There was a negative correlation between the alfalfa and rice straw treatments, while a positive correlation was detected between alfalfa and cornstalk treatments. However, a negative correlation was observed between rice straw and cornstalk treatments, while a low correlation was detected between the two treatments. The untreated control group had shown constant variables.

### 3.3. Volatile Fatty Acid Analyses

The amount of VFA concentration produced after 18 h of the last stage of the in-vitro digestibility experiment is presented in Table 5. There was no significant difference between the different treatment groups in acetic acid concentrations. However, there were significant differences in the propionic acid and n-butyric concentrations. The highest response for VFA was recorded for acetic acid levels for the three treatments, while the lowest response observed for n-butyric acid. Levels of propionic acid and n-butyric acid were significantly higher in rice straw treatment compared to alfalfa treatment. However, no significant difference was observed in levels of propionic acid and n-butyric acid between cornstalk and control treatment. 

VFA peak areas were slightly similar to VFA concentrations, as depicted in Table 6. The alfalfa treatment was significantly different from corn stalk, rice straw, and controls for propionic acid concentrations. However, there was no significant difference in peaks and concentrations of propionic acid for corn stalk and rice straw treatments. Regarding, n-butyric acid, the control had a higher peak area than treatments, and alfalfa treatment differed significantly from rice straw and corn stalk treatments, and controls.

### 3.4. DNA Sequencing, OTU Division, and Classification Status

**Figure 1:**
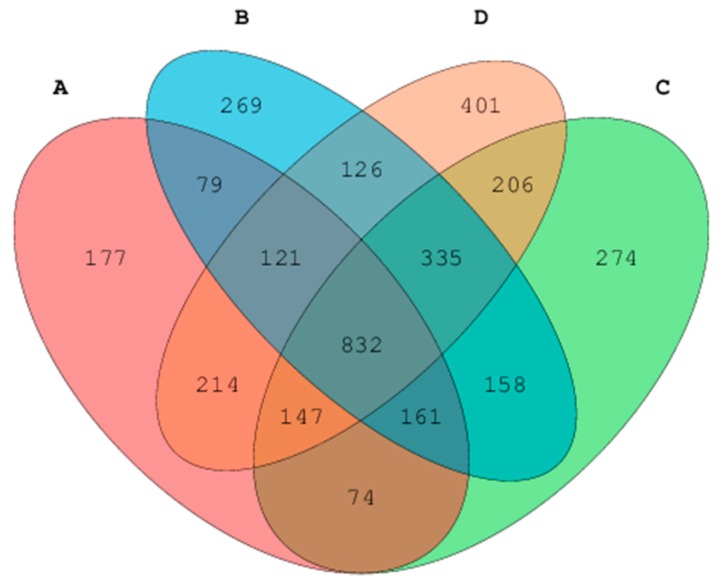
Venn diagram showing shared and unshared operational taxonomic units. A: alfalfa, B: cornstalk, C: rice straw, and D: controls. Note: Each ellipse represents a group of samples corresponding to the three different treatments and the untreated controls.

In the 16s rRNA study, all digesta-liquid samples (*n* = 16) produced 562,047 high-quality sequences with an average of 35,127.9 sequences per sample. A number of OTUs shared and unshared by each sample and sample groups were 2453 and 1121, respectively (Figure 1). The numbers of shared and unshared OTUs for alfalfa treatment were 1628, and 177, respectively. The numbers of shared and unshared OTUs by cornstalk treatment were 1812 and 269, respectively.

The overlapping areas between the ellipses indicate common OTU between sample groups from different treatments. Each block number indicates shared/common or unshared/unique OTUs among treatment groups.

The rice straw treatment shared a high number of OTUs (1913) in comparison to alfalfa and cornstalk treatments, while alfalfa treatment had the lowest number of unshared OTUs. Based on the nucleotide sequence, a minimum of 27 and maximum of 1203 OTUs obtained (Table 7) in this study, while the overall number of OTUs was 72,633 (Table 7).

### 3.5. Alpha Diversity

**Figure 2:**
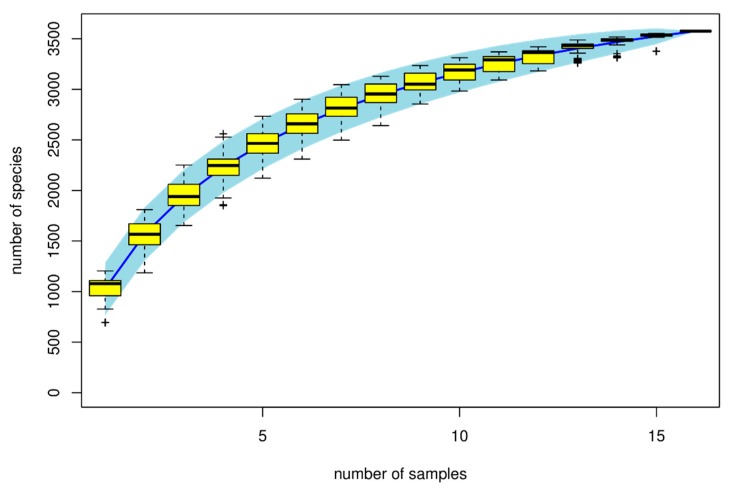
Speccacum species curve. The abscissa represents the sample size, the ordinate represents the number of species being detected, and the blue shade reflects the confidence interval of the curve.

**Figure 3:**
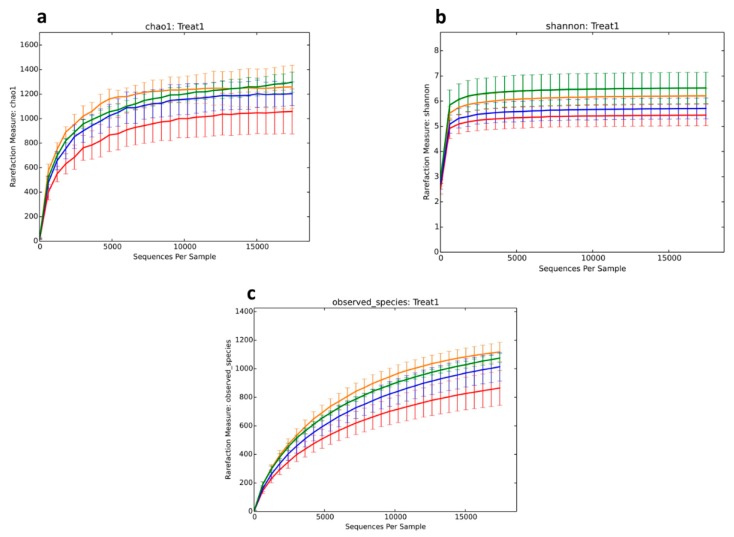
Alpha diversity rarefaction plots of Chao1, Shannon, and observed species. Note: red = alfalfa, blue = cornstalk, orange = rice straw, and green = control. The abscissa represents the total number of randomly extracted sequences in each sample, and the ordinate represents the number of OTUs observed at the corresponding depth. The length of the curve reflects the amount of sample sequencing. The longer the curve, the higher the sequencing depth and the greater the possibility of observing higher diversity.

Rarefaction curves based on the sequencing depth of each sample are shown in Figure 2 and Figure 3 to demonstrate whether the current sequencing was sufficient to reflect the microbial diversity in the sample. The results demonstrate that the number of bacterial species increased as the sample size increased until they reached the optimal level. Alpha diversity measurement (Figure 3), such as the Chao1 index, shows that alfalfa and corn stalk treatments had a significant reduction in richness when compared to the control group and rice straw treatment (Figure 3a). However, the Shannon index shows that the bacterial diversity was significantly affected by the treatments, with rice straw treatment showing higher richness in bacterial diversity in comparison to alfalfa and cornstalk treatments (Figure 3b). The results on observed species showed similar results as the Chao1 index results, and that the sample size was sufficient to reflect the richness of the bacterial community (Figure 3c). Species accumulation curve results (Figure 2) reflect the rate of increase in new bacterial species observed during the continuous and overall sampling period. The results demonstrate that the number of species increased as the sample size increased until they reached the saturation level.

### 3.6. Taxonomic Composition Analysis

**Figure 4:**
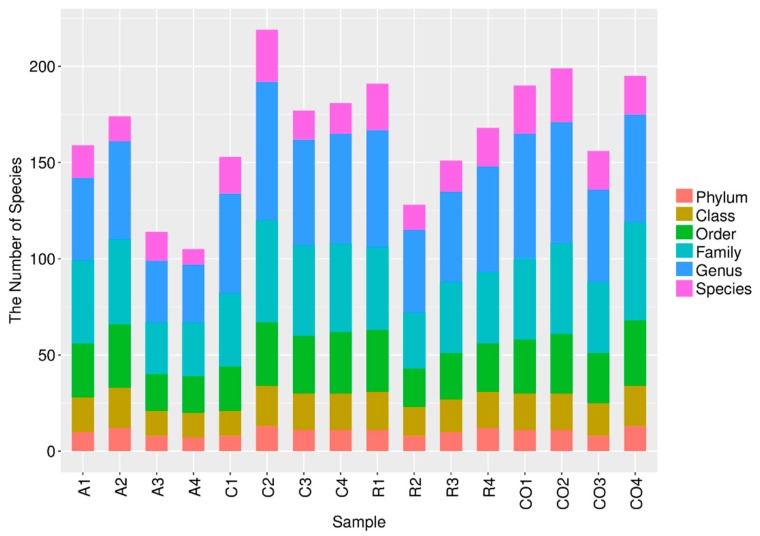
Microbial populations at each classification level. Note: The abscissa depicts the sample name, and the ordinate shows the number of microbial groups in each of the six classification levels, including the classification levels of phylum, class, order, family, genus, and species. (A: alfalfa, C: cornstalk, R: rice straw, CO: control).

The different classification levels in Figure 4, consisting of phylum, class, order, family, genus, and species, demonstrate the bacterial community composition at different resolutions. 

The numbers of microbial groups were compared in samples from different treatments. The results show that phylum level microbial groups were dominant in cornstalk treatment when compared to alfalfa and rice straw treatments. Similar results were discovered in class, family, and genus classification levels. However, the control group showed an increase in the number of species-level microbial groups. Both the cornstalk treatment and control groups showed higher numbers of order-level microbial groups. 

**Figure 5:**
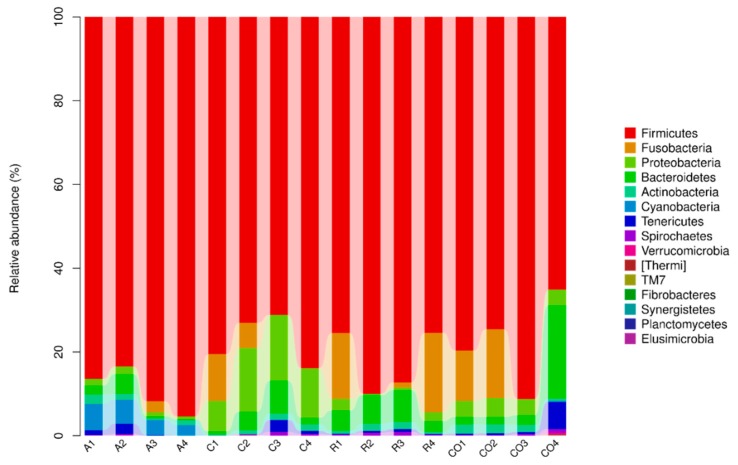
Bacterial community taxonomic composition and abundance at the phylum level. (A: alfalfa, C: cornstalk, R: rice straw, CO: control).

**Figure 6:**
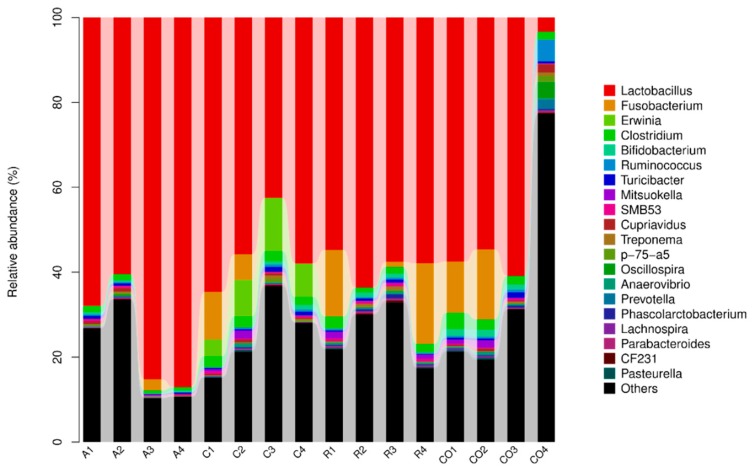
Bacterial community composition and abundance at the genus level. Note: The abscissa is arranged according to the sample name. Each column chart represents a sample, and each classification unit is distinguished by color. The ordinate represents the relative abundance of each classification unit. (A: alfalfa, C: cornstalk, R: rice straw, CO: control).

The bacterial composition and abundance of each sample at the five classification levels were obtained, as depicted in Figure 5. Eight phyla, including, *Firmicutes*, *Fusobacteria*, *Proteobacteria*, *Bacteroidetes*, *Actinobacteria*, *Cynobacteria*, *Tenericutes*, and *Spirochaetes* were the most abundant phyla in all samples. *Firmicutes, Proteobacteria,* and *Actinobacteria* were prominent phyla in the treatment groups. *Firmicutes* were the most abundant in alfalfa treatment in comparison to cornstalk and rice straw treatments (Bacteria: p-*Firmicutes* = 89.2%). The class *Bacilli* was represented with high abundance, and the treatment effect was observed in all treatment groups. *Bacilli, Clostridia, Bacteroidia, Gamaproteobacteria,* and *Actinobacteria* were the dominant classes in all samples, whereas *Actinobacteria* was the least abundant in the alfalfa treatment. At order level, *Lactobacillales, Clostridiales, Bacteroidales, Enterobacteriales, Bidifobacteriales*, and *Erysipelotrichales* were the most abundant orders. However, *Erysipelotrichales* was the least abundant order in all treatment groups, whereas *Lactobacillales* was the predominant order in all treatment groups. Treatment effects were observed in *Lactobacillaceae, Ruminococcacea*, unclassified *Clostridiales, Clostridiaceae, Peptostreptococcaceae*, unclassified *Bacteriodales, Bifidobacteriaceae,* and *Erysipelotrichaceae* families. The less abundant families were *Verrucomicrobiaceae, Leuconostocaceae, Spirochaetaceae, Prevotellaceae,* and *Comamonadaceae*, and rice straw treatment showed less effect (0.2%) in *Prevotellaceae* and *Comamonadaceae* families. At genus level, *Lactobacillus*, unclassified *Ruminococcaceae*, unclassified *Clostridiales*, unclassified *Peptostreptococcaceae,* unclassified *Bacteroidales, Clostridium*, and unclassified *Clostridiaceae* were the most abundant genera (Figure 6). *Lactobacillus*, unclassified *Ruminococcaceae*, and unclassified *Peptostreptococcaceae* were the prominent genera in all treatment groups. The results showed that *Lactobacillus* was most abundant in the alfalfa treatment in comparison to cornstalk and rice treatments (75.2%). The less dominant genera were *Prevotella, Phascolarctobacteroides, Parabacteroides, Ochrobactrum*, and unclassified *Commenadaceae.*

**Figure 7:**
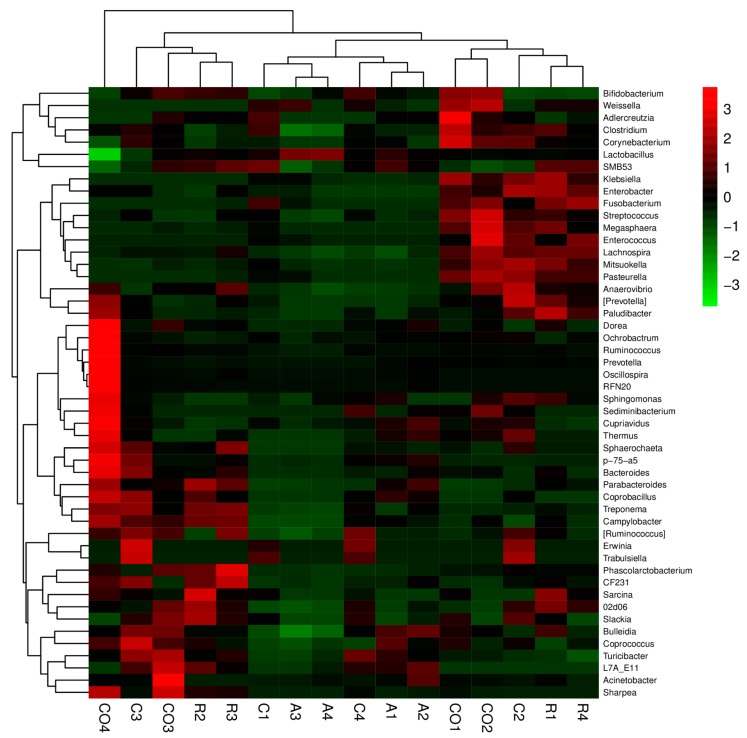
Heat map combined with cluster analysis of bacterial community composition at genus level. (A: Alfalfa, C: Corn stalk, R: Rice straw, CO: Control). The color intensity shows the percentage of bacterial community in a sample at the genus level. In this figure, red represents the genus with higher abundance in the corresponding sample, and green represents the genus with lower abundance.

The results in Figure 7 showed that the bacterial community composition data at each classification level were clustered according to the abundance distribution of the classification unit or the degree of similarity between the samples. The classification unit and samples were sorted according to the clustering result, and presented by the heat map.

### 3.7. Beta Diversity

**Figure 8:**
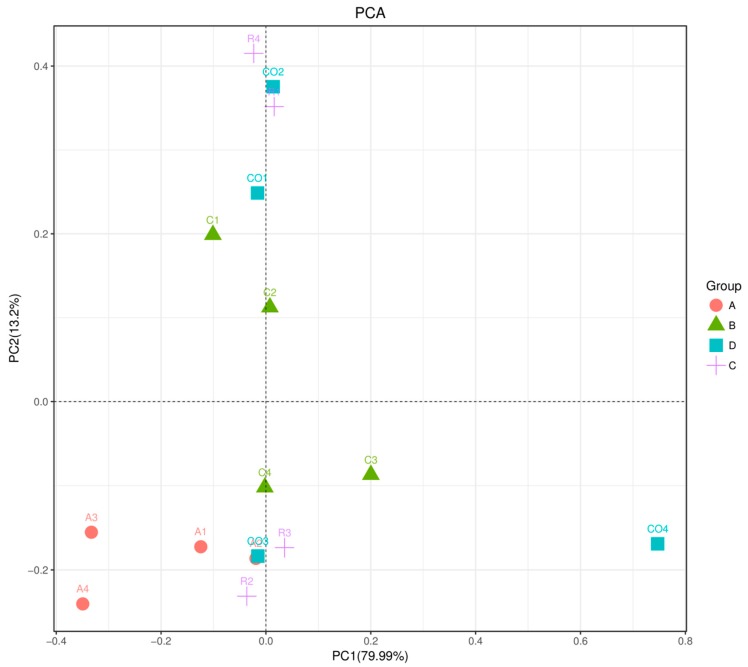
Samples in a two-dimensional sorting graph in PCA analysis. Note: Each point represents a sample. Points of different colors belong to different samples (groups). The closer the distance between the two points, the higher the similarity of microbial community structures between the two samples, and the smaller the difference. The percentage in parentheses in the axis represents the proportion of the difference in the raw data that the corresponding principal component can interpret. (A: alfalfa, C: cornstalk, R: rice straw, CO: control).

The beta diversity index was analyzed by principal component analysis (PCA). Results in Figure 8 show that treatments had high evenness and similarity in the microbial communities as compared to the control group. However, the rice straw treatment group demonstrated microbial community diversity compared to alfalfa and cornstalk treatment groups.

### 3.8. Comparative Analysis of Bacteria

**Figure 9:**
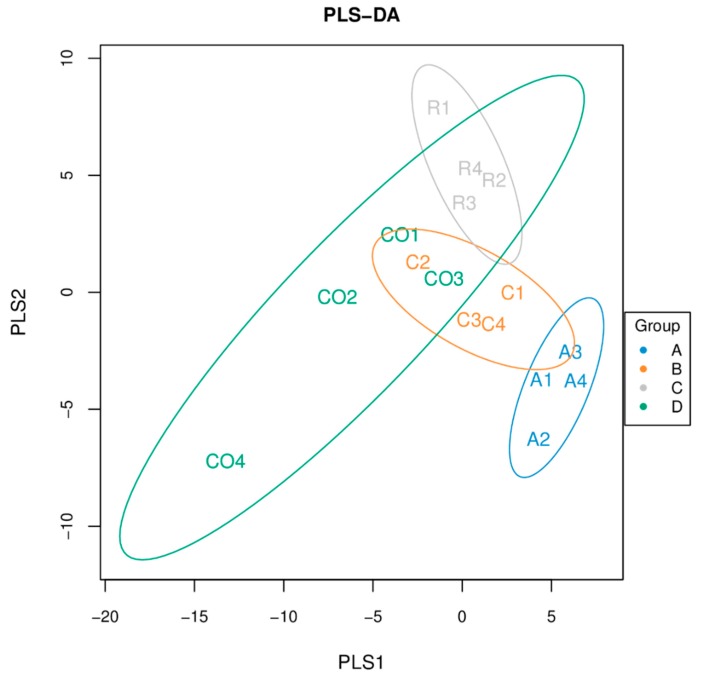
Partial least square discriminant analysis (PLS–DA) chart. Note: Each point represents a sample, the points with the same color belong to the same group, and the points of the same group are marked with an eclipse. If the samples belonging to the same group are closer to each other and the distance between the points of different groups is farther, the better the classification model. (A: alfalfa, B: cornstalk, C: rice straw, D: control).

Comparative analysis of bacteria and screening of key species were analyzed using partial least square discriminant analysis (PLS–DA). Figure 9 demonstrates that the control group (D) shared bacterial species with the rice straw (C) and cornstalk (B) treatment groups, whereas the alfalfa (A) treatment group only shared bacterial species with the corn stalk treatment group. A better classification model was observed between group A and group C and among group A species.

### 3.9. Bacterial Metabolism

**Figure 10:**
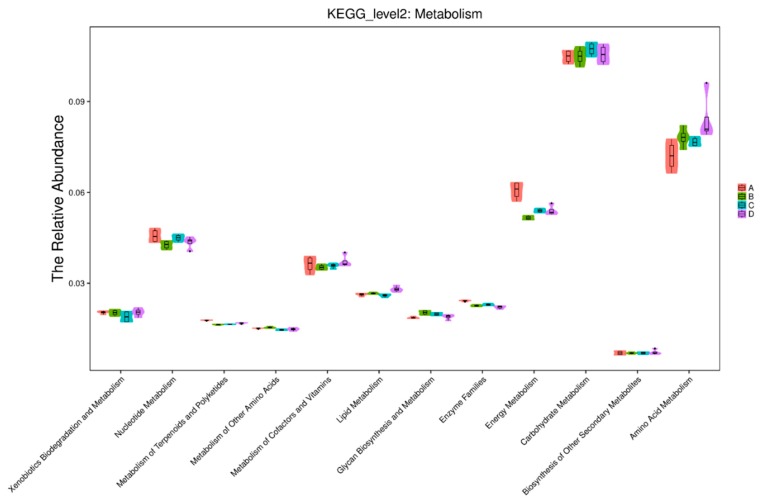
Phylogenetic investigation of communities by reconstruction of unobserved states (PICRUSt) predicted Kyoto Encyclopedia of Genes and Genomes (KEGG) second level distribution map (metabolism). Note: The abscissa is the KEGG second-level functional group, and the ordinate shows the relative abundance of each functional group in each sample (group). A: alfalfa, B: cornstalk, C: rice straw, D: control.

Prediction of bacterial metabolism was analyzed using phylogenetic investigation of communities by reconstruction of unobserved states (PICRUSt) functional predictive analysis. The PICRUSt predicted Kyoto Encyclopedia of Genes and Genomes (KEGG) second level (metabolism) distribution map results are displayed in Figure 10. The carbohydrate metabolism function group had high relative abundance in all treatment groups (0.11), and the relative abundance was lower at a rate of 0.01 for the biosynthesis of other secondary metabolites in all treatment groups. Results on amino acid metabolism showed that more samples from the control group corresponded to the relative abundance, whereas alfalfa group had a higher interquartile range; however, the relative abundance was lower. Energy metabolism function results show a higher relative abundance on alfalfa as compared to other rice straw and cornstalk treatments, and alfalfa also had a higher interquartile range than other treatments.

## 4. Discussion

This study shows that the in-vitro digestibility of the three fiber sources significantly increased in DM% compared to the control group. Cornstalk showed a higher value of digestibility compared to alfalfa and rice straw. Greater digestibility of corn stalk than rice straw and alfalfa in these results may be due to the lower crude fiber content in corn stalk compared to alfalfa and rice straw. Similarly, Park et al. [26] observed that in-vitro digestibility significantly increased in corn and wheat treatments. The levels of fiber in pig’s diet were reviewed as an important element affecting digestibility, and fiber-containing diets have a potential to increase fecal output [27]. In the current study, in-vitro digestibility results for both methods used did not show any effect on the control, while a positive correlation was observed between alfalfa and corn stalk treatment (*p* < 0.01). However, there was a negative correlation between rice straw and cornstalk treatments.

A higher response was observed in all treatment groups in the intestinal digesta method in comparison to the 3-step procedure, which is likely due to the extent of feed residues that were available in the intestinal digesta. Similar results were observed by Chen et al. [28] when the 2-step in-vitro gas production technique (2 + IVGPT) and 3 steps were used, the 2 + IVGPT method presented a significantly higher dietary fiber digestibility compared to the 3-step method. The 3-step method stimulated the hydrolysis of intestinal microbes, which was induced by fibrolytic enzymes mainly and not from fermentation [28,29]. In-vitro fermentation of different fiber treatments resulted in the production of short-chain fatty acids (SCFA), including acetic, propionic, and n-butyric acids that were measured. Rice straw showed a higher concentration of acetic, propionic, and n- butyric acids compared to alfalfa and cornstalk treatments and control. Similarly, Fernando et al. [30] suggested that all probiotic strains on rice fiber formed SCFA, especially acetate, at twice the level of the untreated control. Several studies have demonstrated that different fiber sources are fermented at different rates and produce different amounts of SCFA [31]. Our results show that the fermentation rates are clearly dependent on the fiber source; hence, the concentration rates and peak areas differ. Acetic acid concentration and peak area were significantly higher compared to other SCFA; however, there no significant difference detected between different treatments The higher concentration of SCFA in dietary fiber shows that the substrate in rice straw contains more fermentable material than other treatments [30]. In the current study, it was observed that peak areas were significantly higher in the controls than treatment groups in all measured SCFA. The alfalfa treatment demonstrated a significant decrease in SCFA concentrations and peak areas in contrast to findings by Liu et al. [32], who indicated that alfalfa meal treatment could produce more SCFA and improve piglet gut health, which was associated with less diarrhea. This may be dependent on the age of the pig and the level of feeding. All treatments and untreated controls showed less effect on n-butyric concentrations and peak areas. Previously, butyric acid had a high response on organic acids when used as feed additives. Gut microbiota contains numerous and diverse microbial populations [33], and in the past, the identification of porcine microbiota has been made using culture-dependent techniques.

However, the knowledge of microbiota has advanced over the years with the advent of next-generation sequencing technology and bioinformatics [34]. The effect of dietary fibers on bacterial composition during fermentation was investigated using 16S rRNA based methods and illumina MiSeq sequencing. OTU analysis in this study revealed microbial diversity in cecum digesta in all treatment groups. We found that alpha diversity indices differed based on treatments in terms of species richness and evenness. The Chao1 index showed that there was less microbial diversity among treatments compared to controls, whereas microbial richness and species diversity increased among the treatments, as shown by the Shannon diversity index. In agreement with Yu et al. [35], who showed similar results in the cecum microbial population with chitosen supplementation. Results of the speccacum species curve in Figure 3 show that the number of microbial species increases as sample size increases, until they reach optimal level. Microbial composition classification on each classification level showed a high number of bacterial species at the genus level and Lactobacilli were the most abundant bacterial genus. *Lactobacillus* are considered probiotics and have been associated with the promotion of gastrointestinal tract health [36]. Lactobacilli prevent infections and colonization of the gut by pathogens and produce antimicrobial factors, including bacteriocins and lactic acids [37]. Niu et al. [38] found that Lactobacillus was the abundant genera accounting for more than 84% of total sequences. This study revealed that *Firmicutes*, *Bacteriodetes*, *Proteobacteria*, *Cynobacteria,* and *Actinobacteria* were the dorminant bacterial phyla. Furthermore, alfalfa treatment was associated with significant Firmicutes abundance, which accounted for 89.2% sequences. Alfalfa diet has been associated with the ability to decrease the number of pathogenic bacteria and an increase in the number of bacteria that promote alfalfa fiber hydrolysis [39]. In this study, all treatments and the control showed less effect on the abundance of *Prevotella*, *Phascolarctobacteroides*, *Parabacteroides*, *Ochrobactru,* and unclassified *Commenadaceae* genera. Previously, other studies revealed that *Prevotella* and *Bacteroidetes* genera and phylum were abundant in the gut when fibrous diets were used [40]. In this study, only *Bacteroidetes* were abundant, while marginal levels of *Prevotella* were observed. However, our results concur with Gorvitovskaia et al. [41], who showed that there was an inverse correlation between of Bacteriodes and Prevotella: when *Bacteroides* numbers were high in a sample, *Prevotella* was less abundant and vice versa. Liu et al. [42] stated that *Prevotella* species promote fiber, pectin, protein, and hemicellulose utilization processes and enhance low pH in the ruminal environment caused by sub-acute ruminal acidosis and reduced numbers of *Prevotella*. There was a significant difference between all taxonomic units. However, *Gammaproteobacteria*, *Enterobacteriales*, *Enterobacteriaceae*, *Proteobactera,* and *Erwina* showed high significance with a 4.7 to 4.8 LDA score (log10) for cornstalk treatment. Principal component analysis (PCA) of microbial evenness and diversity revealed a significant decrease in microbial community diversity among all dietary fiber treatments and marginal diversity in the controls. The three treatments were effective in assessing microbial diversity. Furthermore, rice straw treatment group showed microbial community diversity compared to alfalfa and cornstalk treatment groups, and microbial functions were more available on alfalfa treatment in comparison to other treatments. Nevertheless, the results were based on a limited sample size, and larger well-designed studies are expected to confirm these preliminary findings. Thus, further investigations should be conducted in this line using higher sample sizes. Several factors, such as age, genetic factors, development of diseases, and diet affect the gut microbial community. In this study, the age, breed, previous diet fed, and sex of pigs were not considered.

## 5. Conclusions

In conclusion, corn stalk dietary fiber improved dry matter digestibility in simulated in vitro digestion experiments, whereas rice straw fiber improved volatile fatty acid content in simulated in vitro digestion and fermentation efficiency. Furthermore, alfalfa fiber improved the thickness of *Firmicutes* and *Lactobacillus* supernatant in simulated in vitro digestion treatments.

## Figures and Tables

**Table 1:** Feed sample composition for experimental diets.

Components and Ingredients	Alfalfa Hay (%)	Corn Stalk (%)	Rice Straw (%)
**CF**	34.7	32.7	35.4
**CP**	11.36	2.47	3.25
**EE**	1.63	3.1	0.72
**Ash**	8.42	8.46	10.72
**Moisture**	8.65	10.52	7.49
**NFE**	35.24	42.73	42.42

Note: CF, crude fiber; CP, crude protein; EE, ether extract; NFE, nitrogen free extract.

**Table 2:** In vitro dry matter digestibility; means and standard errors.

Treatments	DM%	Response, %
Alfalfa	19.72 ± 0.63283 ^b^	53.70 ± 1.238 ^b^
Rice straw	18.36 ± 0.43640 ^b^	51.49 ± 2.62 ^b^
Corn stalk	22.61 ± 2.06709 ^a^	57.16 ± 4.22 ^a^
Untreated control	0.000 ± 0.000 ^c^	0.00 ± 0.00 ^c^

Means with different superscript letters are significantly different (*p* < 0.01).

**Table 3:** In-vitro dry matter digestibility; Pearson correlation.

Treatments	Alfalfa	Rice Straw	Corn Stalk	Untreated Control
Alfalfa	1.00			
Rice straw	−0.033	1.00		
Corn stalk	0.984	0.148	1.00	
Untreated control	A	A	A	A

A, cannot be computed because at least one of the variables is constant. *p* < 0.01.

**Table 4:** In-vitro digestibility on digesta; Pearson correlation.

Treatments	Alfalfa	Rice Straw	Corn Stalk	Control
Alfalfa	1.00			
Rice straw	−0.464	1.00		
Corn stalk	0.915	−0.067	1.00	
Untreated control	A	A	A	A

A, cannot be computed because at least one of the variables is constant (*p* < 0.01).

**Table 5:** Volatile fatty acid concentration (mmol/L).

Treatment	Acetic Acid	Propionic Acid	n-Butyric ACID
Alfalfa	53.70 ± 9.33 ^a^	11.83 ± 2.21 ^b^	6.58 ± 1.63 ^b^
Cornstalk	78.09 ± 4.36 ^a^	17.86 ± 0.88 ^ab^	11.04 ± 0.77 ^ab^
Rice straw	83.92 ± 6.59 ^a^	22.11 ± 1.43 ^a^	14.99 ± 1.06 ^a^
Untreated control	65.62 ± 17.18 ^a^	16.55 ± 4.63 ^ab^	9.90 ± 2.77 ^ab^

Means with different superscript letters in the same column are significantly different *(p* < 0.05).

**Table 6:** Volatile fatty acid peak area.

Treatments	Acetic Acid	Propionic Acid	n-Butyric Acid
Alfalfa	623.00 ± 1.08 ^a^	290.1 ± 54.1 ^b^	240.8 ± 59.5 ^b^
Corn stalk	906.50 ± 50.6 ^a^	437.8 ± 21.6 ^ab^	403.9 ± 28.3 ^ab^
Rice straw	762.00 ± 1.99 ^a^	406.00 ± 1.13 ^ab^	363.00 ± 1.01 ^a^
Untreated control	974.10 ± 76.5 ^a^	542.10 ± 35.1 ^a^	548.6 ± 38.6 ^a^

Means with different superscript letters in the same column are significantly different *(p* < 0.05). Values are means with the standard error.

**Table 7:** Operational taxonomic units classification and classification status identification results.

Name	Number of OTUs
Phylum	16,423
Class	16,423
Order	16,342
Family	13,509
Genus	8988
Species	948
Minimum number per sample	27
Maximum number per sample	1203
Mean per sample	35,127.9
